# Accounting for stillbirths in maternal health metrics: a cross-country analysis

**DOI:** 10.1016/j.eclinm.2025.103303

**Published:** 2025-06-23

**Authors:** Ursula Gazeley, Hallie Eilerts-Spinelli, Joshua Wilde, Hannah Blencowe, Li Liu, Wendy Graham, Veronique Filippi

**Affiliations:** a Leverhulme Centre for Demographic Science, Nuffield Department of Population Health, University of Oxford, Oxford, United Kingdom; b Department of International Health, Johns Hopkins Bloomsberg School of Public Health, Baltimore, MD, United States; c Portland State University, Portland, OR, United States; d Department of Infectious Disease Epidemiology and International Health, London School of Hygiene and Tropical Medicine, London, United Kingdom; e Department of Population Family and Reproductive Health, Johns Hopkins Bloomsberg School of Public Health, Baltimore, MD, United States

**Keywords:** Maternal mortality, Maternal health metrics, Stillbirth, Pregnancy loss

## Abstract

**Background:**

Live birth is only one of four potential pregnancy outcomes, alongside stillbirth, miscarriage, and induced abortion. While morbidity and mortality associated with all pregnancy outcomes are included in the numerator of many maternal metrics, often only live births are included in the denominator. This inconsistency makes interpreting trends challenging and may exacerbate the deprioritisation of monitoring other pregnancy outcomes. We assess the effect of using (1) total births (live births and stillbirths) or (2) total pregnancies (total births plus miscarriages/induced abortions) as the denominator on estimates of maternal and pregnancy-related mortality ratios (MMR and PRMR).

**Methods:**

Using data from Demographic and Health Surveys (DHS) conducted from 1996 to 2023, we estimated the proportion of pregnancies reported to end in live birth, stillbirth, or miscarriage/induced abortion in full pregnancy histories (DHS-VIII) or reproductive calendars (DHS-VII and earlier) for 46 countries in Africa, Asia, Latin American and the Caribbean, and Oceania. We calculated MMR and PRMR from the DHS sibling survival histories, adjusting the denominator by the reported distribution of pregnancy outcomes to account for either total births or total pregnancies.

**Findings:**

There was substantial cross-country heterogeneity in the proportion of pregnancies reported as ending in a live birth, ranging from 70% (Cambodia 2021) to 96% (Papua New Guinea 2017). Pregnancies reported as ending in stillbirth ranged from 0.3% (Timor-Leste 2016) to 4.1% (Lesotho 2014). Variability across countries might reflect differences in the distribution of pregnancy outcomes, temporal trends, and reporting practices. These differences result in non-uniform biases from using live births as the denominator. Using total births reduced the MMR and PRMR by up to 2.8% (Cote-d'ivoire 2021). Using total pregnancies reduced the MMR and PRMR by up to 23% (Cambodia 2021).

**Interpretation:**

Pregnancy-related morbidity and mortality can occur with any pregnancy outcome, not only live births. Progress in the availability of global stillbirth estimates means using total births as the denominator in maternal metrics is increasingly feasible in some countries and, in turn, could further strengthen momentum to institutionalise stillbirth reporting in civil registration systems. As the end of the SDG era approaches, the use of a more conceptually accurate maternal denominator based on total births should be explored in parallel with existing measures. However, better estimates of miscarriage and induced abortion are needed before total pregnancies can be used in global maternal metrics.

**Funding:**

UG and JW were supported by the 10.13039/100014013UK Research and Innovation (EP/Y031172/1) and the 10.13039/501100000275Leverhulme Trust (RC-2018-003) for the Leverhulme Centre for Demographic Science. HES and LL were supported by the 10.13039/100009633Eunice Kennedy Shriver National Institute of Child Health and Human Development (R01HD107015) and the Gates Foundation Child and Adolescent Causes of Death Estimation (CA-CODE, INV-038624).

**Research in context**

**Evidence before this study**

Maternal metrics have historically relied on live birth denominators due to challenges in capturing other pregnancy outcomes and the availability of routine data. We conducted a search of Embase, MEDLINE, and Global Health (30 April 2025) for English-language studies, with no date restrictions, that analyse the effect of using live birth denominators in maternal metrics (for title and abstract search terms see Appendix p.2). The limited existing evidence suggested that using a total pregnancy denominator instead of live births may lower the Maternal Mortality Ratio (MMR) by accounting for all pregnancy outcomes.

**Added value of this study**

We provide the first cross-country estimates of the impact of using live birth denominators using DHS data from 46 low- and middle-income countries (LMICs) from 1996 to 2023. Adjusting the denominator of the MMR from live births to (1) total births or (2) total pregnancies led to average reductions of 1.3% and 9.4%, respectively. However, heterogeneity in pregnancy outcomes, and heterogeneity in the reporting of these outcomes, means the extent of bias varies globally: using total births reduced the MMR by up to 3% (Côte d’Ivoire 2021), while using total pregnancies reduced the MMR by up to 24% (Cambodia 2021).

**Implications of all the available evidence**

Persistent challenges in the measurement of early pregnancy loss and induced abortion in routine civil registration and vital statistics (CRVS) and facility-based data currently hinder the adoption of a total pregnancy denominator. However, substantial progress in stillbirth estimation in recent decades means using a total birth denominator is increasingly viable. Metrics using a total birth denominator could accompany conventional measures to provide different insights and support interpretation, strengthening global momentum to improve stillbirth surveillance and increase the visibility of stillbirths in the maternal health agenda.

## Introduction

Many pregnancies do not end in a live birth. Live birth is one of four possible outcomes, along with miscarriage, stillbirth, and induced abortion, including those following ectopic and molar pregnancies. Complications associated with each outcome may lead to maternal morbidity or mortality, meaning all pregnancies should be counted when assessing risk. However, live births were originally chosen as a reasonable proxy for the denominator of maternal health metrics due to challenges in capturing other pregnancy outcomes. Most notably, live births are the denominator of the Maternal Mortality Ratio (MMR: maternal deaths per 100,000 live births). The MMR is the metric used in Sustainable Development Goal 3.1: to reduce the global MMR to below 70 before 2030.^1^ This target is used to track progress between countries and over time.^2^

Using live births as the denominator introduces problems, illustrated with the MMR. First, it creates an incongruence between the numerator and the denominator at risk.^3^ Maternal deaths are defined in the International Classification of Diseases (ICD)-11 as “*the death of a woman during pregnancy or within 42 days of termination of pregnancy, from any cause related to or aggravated by pregnancy or its management, excluding accidental or incidental causes*”.^4^ Deaths following pregnancies that do not end in a live birth are included in the numerator but not the denominator, inflating the indicator.^3^ Complications related to pregnancies with abortive outcome, including induced abortion, miscarriage, and ectopic pregnancy, are a significant contributor to maternal mortality, accounting for 8% of all maternal deaths worldwide.^5^ In settings with limited access to safe abortion services, abortion-related morbidity is also substantial, with 9% of abortion-related hospital admissions experiencing a life-threatening maternal near miss complication.^6^ Globally, the WHO estimates there are around 73 million induced abortions annually,^7^ and approximately 45% are unsafe.^8^ Excluding non-live birth outcomes from the denominator reinforces the misconception that maternal deaths are only consequent to live births and obscures the substantial risks from other pregnancy outcomes.^3^

Second, as the MMR is currently defined, the mismatch between the numerator and denominator means that changes may reflect either fluctuations in the number of maternal deaths or live births. Transitions in stillbirth and maternal mortality from high to low levels are highly correlated, and interventions to reduce maternal risk may simultaneously reduce stillbirths and increase live births.^9^ However, using live births in the denominator can create biases when comparing settings where rates of pregnancy loss vary significantly, often due to differences in access to- and the quality of-maternal and reproductive health services. Such differences are also closely linked to variation in the ascertainment of pregnancy outcomes, and the quality and completeness of birth registration data, further complicating cross-country comparisons.

Third, the inclusion of only live births in the denominator of the MMR deprioritises measurement of outcomes from pregnancies ending in miscarriage, stillbirth, or abortion.^3^ Decisions regarding what to measure and how have important implications for the maternal health agenda. Indicators govern public health priorities, influencing resource allocation, health interventions, and policy design.^10^^,^^11^ Historically, measurement of induced abortions, miscarriages and stillbirths have received less attention, and challenges in measuring these outcomes through routine health facility data and civil registration and vital statistics (CRVS) systems have limited the completeness of pregnancy outcome data. However, considerable progress has been made to improve stillbirth surveillance in recent years,^12^^,^^13^ although challenges remain in many low- and middle-income countries’ (LMICs) CRVS.^12^^,^^14^^,^^15^ Incomplete coverage of CRVS systems, incomplete registration of live births and stillbirths, and misclassification between stillbirths and early neonatal deaths persist in many settings.^12^^,^^14^^,^^15^

These problems of using a live birth denominator are not unique to the MMR but affect any maternal indicator using live births.^16^ Examples include the pregnancy-related mortality ratio (PRMR), indicators dependent on the MMR such as the lifetime risk of maternal death, maternal morbidity metrics, and indicators of obstetric interventions (e.g., caesarean section rate).

With just five years left of the Sustainable Development Goal (SDG) era,^1^ now is an opportune time to reconsider the denominator issue and explore alternative approaches. Our objective is to quantify the effect of using (1) total births (live births and stillbirths), or (2) total pregnancies (live births, stillbirths, miscarriages/induced abortions) instead of only live births on the MMR and PRMR across LMICs.

## Methods

### Overview

In this cross-sectional comparative analysis, we recalculated the MMR and PRMR using alternative denominators (total births or total pregnancies). Although this analysis centres on these two key indicators, the implications of using live birth denominators applies broadly across maternal metrics (see Table 1). Denominators were adjusted using the reported distribution of pregnancies ending in live birth, stillbirth, or miscarriage/induced abortion.

**Table 1:** Maternal health indicators used for global maternal health monitoring dependent on a live birth denominator.

Metric	Numerator	Denominator
**Mortality**		
Maternal mortality ratio (MMR)	Maternal deaths	100,000 live births
Pregnancy-related mortality ratio (PRMR)	Pregnancy-related deaths^a^	100,000 live births
Lifetime risk of maternal death (LTR-MD)	MMR as dependency	100,000 live births
Lifetime risk of pregnancy-related death (LTR-PRD)	PRMR as dependency	100,000 live births
**Morbidity/intervention**		
Maternal near miss ratio (MNMRatio)	Maternal near miss events^b^	1000 live births
Severe maternal outcome ratio (SMORatio)	Maternal near miss events and maternal deaths	1000 live births
Lifetime risk of maternal near miss (LTR-MNM)	MNMRatio as dependency	1000 live births
Lifetime risk of severe maternal outcome (LTR-SMO)	SMORatio as dependency	1000 live births
Caesarean section rate^c^^,^^d^	Caesarean deliveries	100 live births
Skilled attendance at birth rate^c^^,^^e^	Births attended by skilled health personnel	100 live births
Antenatal care (ANC) coverage rate^c^^,^^f^	Mothers receiving specified number of ANC visits during pregnancy	100 live births

a A pregnancy-related death is defined as “the death of a woman while pregnant or within 42 days of termination of pregnancy, irrespective of the cause of death (obstetric or non-obstetric)”. This definition includes accidental and incidental causes.^17^

b A maternal near miss event is defined as “a woman who nearly died but survived a complication that occurred during pregnancy, childbirth, or within 42 days of termination of pregnancy”.^18^

c Most, but not all, international data use a live birth denominator. For example, World Health Organization Global Health Observatory data https://www.who.int/data/gho. However, since DHS-VIII, the DHS program have expanded the population-base to include both live births and stillbirths in the (see DHS program for details).

d Stillborn babies born by caesarean section will be included in the numerator but are usually excluded from the denominator.

e Births attended by skilled health personnel (%) with a live birth denominator is SDG indicator 3.1.2.

f ANC coverage with a live birth denominator is a tracer indicator of health services for the universal health coverage SDG indicator 3.8.1.

### Data

For over 40 years, the Demographic and Health Surveys (DHS) have been a cornerstone of global health research, providing nationally representative, internationally comparable, population-level estimates on a wide range of indicators—including maternal and child health, fertility, family planning, HIV, nutrition, and water and sanitation—through large-scale household surveys in LMICs.^19^ The DHS measure maternal and pregnancy-related mortality using the sibling survival module, with mortality rates estimated via the Sisterhood method.^20^ Respondents list their siblings and indicate whether each sibling is alive or dead. For deceased sisters aged 12 or older at the time of death, respondents report whether the death occurred during pregnancy, childbirth, or the postpartum period. Pregnancy-related deaths are defined in the DHS as deaths within two months of the end of pregnancy, irrespective of cause. Since 2015, the DHS define maternal deaths as those occurring during pregnancy, childbirth, and up to 42 days postpartum, excluding deaths due to accidents or violence.^20^ The DHS definition of maternal death is not consistent with the ICD-11 definition, as incidental causes are not excluded.^4^ Due to the statistical infrequency of maternal and pregnancy-related mortality, the DHS include sibling survival modules only once every 10 years.^21^

The DHS have implemented several approaches to pregnancy reporting over time that facilitate disaggregation by outcome type.^22^ Full maternity history questionnaires have been the predominant method utilised by the DHS for pregnancy reporting.^22^ Starting in DHS-I (1984–1990), the model questionnaire included a full birth history (FBH) for reporting of live births only. From DHS-II (1989 onwards), reproductive calendars were used to collect month by month information on contraceptive use and reproductive events, typically for a five-year period prior to the survey for women aged 15–49 years. Calendars facilitated reporting of stillbirths and pregnancies ending before six months’ gestation (miscarriage or induced abortion, not differentiated). Since DHS-VIII (2018 onwards), a full pregnancy history (FPH) has been used to collect data on women’s live births, stillbirths, miscarriages and induced abortions, disaggregated.

Neither the reproductive calendar nor FPH capture pregnancy length in weeks or days, preventing the application of the standard ICD-11 stillbirth definitions (i.e., early foetal deaths from 22 to 28 weeks’ gestation or 500-100 g, and late foetal deaths after 28 weeks or greater than 1000 g).^23^ Stillbirths are instead approximated as pregnancy losses occurring at seven or more months of gestation. The reproductive calendar’s single month entry format cannot capture discordant outcomes in multiple gestations, such as one twin being born alive and the other stillborn.

We identified all surveys since the inception of the DHS program which had a sibling survival module in addition to a reproductive calendar (n = 104) and/or FPH (n = 4) in the analysis. This was necessary to assess maternal/pregnancy-related deaths and pregnancy outcomes, respectively. For each country, we kept their most recent DHS, if more than one survey was available, with surveys from 1996 to 2023 (n = 46). For the calculation of maternal mortality, the analytical sample was further limited to surveys from DHS-VI and onwards which included information regarding whether the sibling’s death was due to violence or an accident (n = 26). These deaths are considered pregnancy-related but are excluded from maternal mortality.^23^ Some DHS surveys only include ever married women in their sample for pregnancy reporting and sibling survival (n = 2). Included surveys and survey characteristics can be found in Appendix Table S1 (Appendix pp.3–4).

### Statistics

All procedures were conducted using R version 4.4.1 and are reproducible from DHS data (registration required).

We used the open-access code from the *DHS.rates* package to calculate the maternal and pregnancy-related mortality ratios (MMR and PRMR), and age-adjusted general fertility rate (GFR) from DHS data.^24^ First, maternal (MD_x_) and pregnancy-related deaths (PrD_x_) were divided by person years of exposure of the siblings (E_Sx_) for five-year age groups from 15 to 49 years (x), for a time period of seven years prior to each survey. This yielded the maternal and pregnancy-related mortality rates (MD_x_/E_Sx_) and (PrD_x_/E_Sx_), respectively. These rates were then weighted by the age distribution of female respondents (C_x_) (accounting for individual sampling weights) and divided by the GFR for the same period to derive the MMR and PRMR, both expressed per 100,000 live births (Equations (1), (2)):

(1) $$MMR=\sum_{x}\frac{\frac{MD_{x}}{E_{Sx}}\;\cdot C_{x}}{GFR}\;\cdot \;100,000$$

(2) $$PRMR=\sum_{x}\frac{\frac{\mathrm{Pr}D_{x}}{E_{Sx}}\;\cdot C_{x}}{GFR}\;\cdot \;100,000$$

The GFR (Equation (3)) was calculated by dividing the number of live births (B_x_) by the person-years of exposure of the respondents (E_Rx_) in each age group, weighted by their age distribution (C_x_), and adjusted using age-specific factors (A_x_) to account for total births or total pregnancies:

(3) $$GFR=\sum_{x}\frac{B_{x}}{E_{Rx}}\;\cdot \;C_{x}\;\cdot \;A_{x}$$

Adjustment factors (A_x_) were derived from each survey’s reproductive calendar or FPH, if available. For each age group, we calculated the distribution of a) live births (LB_x_), b) stillbirths (SB_x_), and c) miscarriages or induced abortions (MSC or AB_x_), reported for the five years preceding the survey, and accounting for sampling weights. Adjustment factors for total births (Equation (4)) and total pregnancies (Equation (5)) were calculated as follows:

(4) $${total\;births\;A}_{x}=\sum_{x}\frac{{LB}_{x}\;+\;{SB}_{x}}{{LB}_{x}}$$

(5) $${total\;pregnancies\;A}_{x}=\sum_{x}\frac{{LB}_{x}\;+\;{SB}_{x}\;+\;{\left(MSCorAB\right)}_{x}}{{LB}_{x}}$$

Following the approach outlined in the *DHS.rates* package, we used the Jackknife method to estimate standard errors for MMR and PRMR, accounting for clusters and sample strata.^24^ We derived 95% confidence intervals using the normal approximation (z = 1.96).

### Ethics

As our analyses used only secondary, fully anonymised DHS data publicly available upon registration, this research did not require ethical approval as per the University of Oxford’s ethical review portal. DHS are available at: https://dhsprogram.com/ and their ethical review statement is available at: https://dhsprogram.com/methodology/Protecting-the-Privacy-of-DHS-Survey-Respondents.cfm.

### Role of the funding source

The funders of the study had no role in study design, data collection, data analysis, interpretation or writing of the report.

## Results

**Fig. 1:**
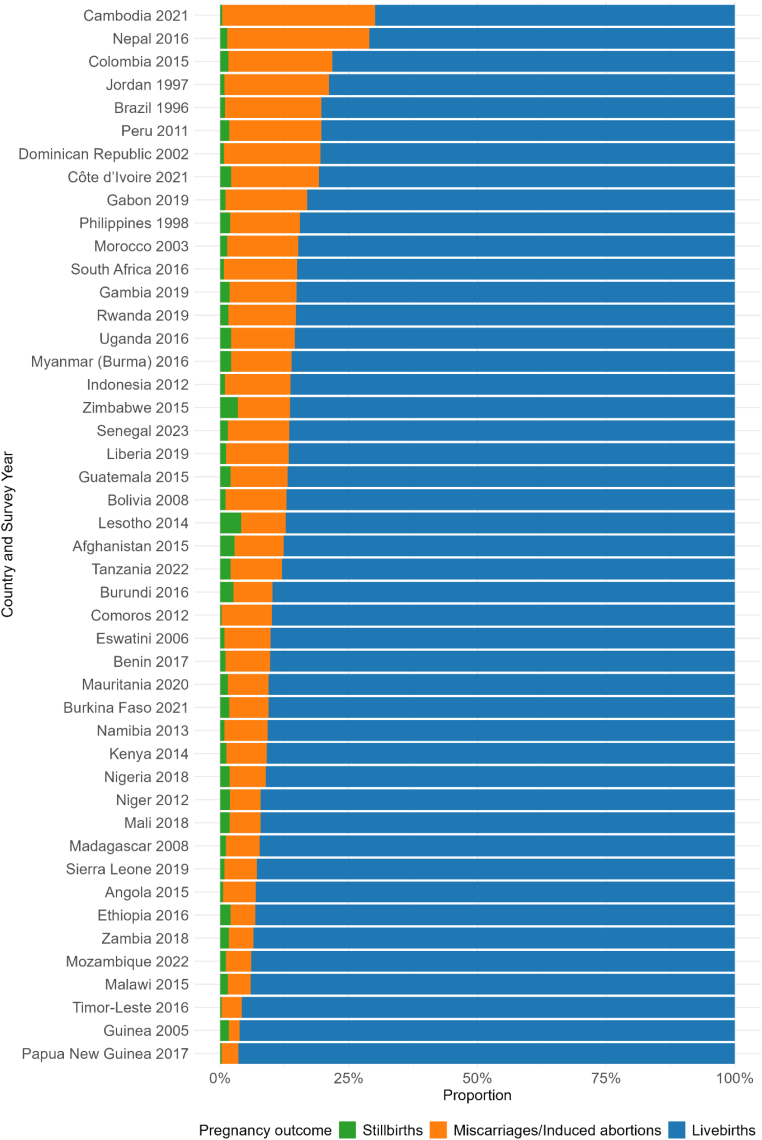
Reported pregnancy outcomes by country, most recent Demographic and Health Survey.

In total, we estimated pregnancy outcome proportions across 46 countries, with survey years from 1996 to 2023. Fig. 1 shows substantial heterogeneity across countries in the reported proportion of pregnancy outcomes from their most recent DHS survey. The mean proportion of live births among total pregnancy outcomes was 87% across countries (range 70% in Cambodia 2021 to 96% in Papua New Guinea 2017). The proportion of pregnancies reported as ending in stillbirth ranged from 0.3% in Timor-Leste 2016 to 4.1% in Lesotho 2014, while miscarriages/induced abortions ranged from 2.2% in Guinea 2005 to 30% in Cambodia 2021.

For the four countries with both a sibling survival module and FPH, the proportion of pregnancies reported as ending in miscarriage ranged from 6.3% in Burkina Faso 2021 to 18% in Cambodia 2021, while the induced abortions ranged from 0.2% in Tanzania 2022 to 14% in Cambodia 2021 (Appendix Fig. S1, p.5).

**Fig. 2:**
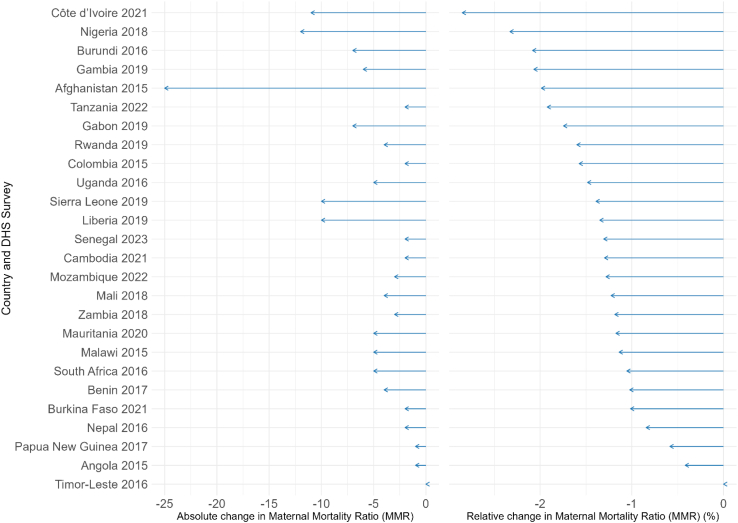
Absolute and relative change in Maternal Mortality Ratio (MMR) using a total birth denominator instead of live births.

In total, we estimated the change in the MMR using alternative denominators for 26 countries with available maternal death data. Fig. 2 shows the impact of applying a total birth denominator on the MMR. The mean relative reduction using a total birth denominator was 1.4%, with the greatest decline observed in Côte d’Ivoire (2021) at 2.8%. The largest absolute difference was in Afghanistan (2015), at −25 per 100,000.

Using a total pregnancy denominator, the mean relative reduction in the MMR was 9.3%, with the greatest decline observed in Cambodia 2021 (−23%) at 23.4%. The largest absolute difference was again in Afghanistan, at −128 per 100,000 (see Appendix Fig. S2, p.6).

**Fig. 3:**
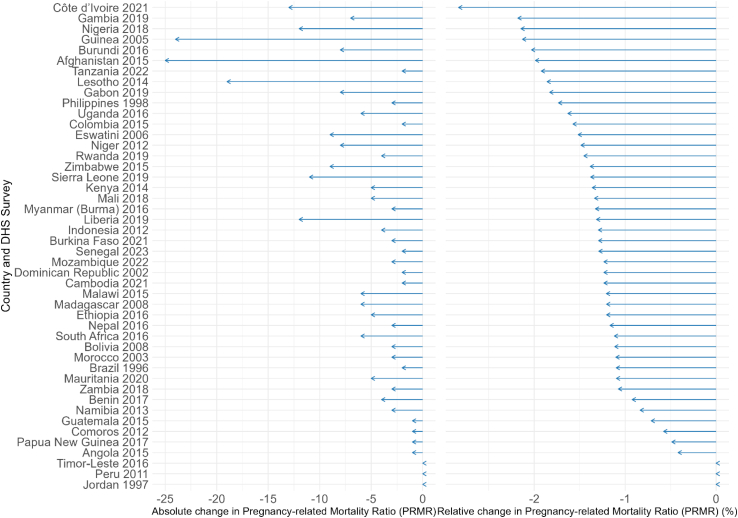
Absolute and relative change in Pregnancy-related Mortality Ratio (PRMR) using a total birth denominator instead of live births.

In total, we estimated the change in the PRMR using total births or total pregnancies as the denominator for 46 countries with available data on pregnancy-related deaths. Fig. 3 shows the impact of applying a total birth denominator on the PRMR. The relative reduction in the PRMR with a total birth denominator was greatest in Côte d’Ivoire 2021 (−2.8%).

The reduction in the PRMR was much greater when using a total pregnancy denominator, with the largest relative decrease observed in Cambodia 2021 (−23%) (see Appendix Fig. S3 p.7). Full results for MMR and PRMR calculations with alternative denominators are provided in Appendix Table S2 (p. 8) and Appendix Table S3 (pp.9–10), respectively.

Finally, confidence intervals for the re-calculated MMR and PRMR were wide, suggesting substantial uncertainty in DHS estimates of maternal and pregnancy-related mortality (see Appendix Figs. S4 and S5 pp.11–12). Estimates using a total births and total pregnancies denominator rather than live births fell within the uncertainty bounds associated with the MMR and PRMR.

## Discussion

To our knowledge, we present the first cross-country comparison of the implications of using live births as the denominator in maternal health metrics. Women are at risk of experiencing adverse outcomes during and consequent to pregnancies not ending in a live birth. However, due to difficulties in the estimation of stillbirths, miscarriages, and induced abortions, live births have been used as a reasonable proxy for the population at risk of maternal morbidity and mortality. Yet the exclusion of these other pregnancy outcomes from the denominator of many maternal metrics has, to date, received limited scrutiny—especially in many low-income contexts where the burden of maternal morbidity and mortality is highest.

Using maternal and pregnancy-related mortality ratios as illustrative examples, our results underscore the impact of this measurement decision. Across 46 countries with DHS surveys from 1996 to 2023, we find that live births accounted for 70–96% of reported pregnancies. There was substantial heterogeneity in the reported proportion of pregnancies ending in stillbirth (0.3%–4.1%) and miscarriage/induced abortion (2.2%–30%). Differences across DHS surveys may reflect genuine cross-country variation and temporal changes in pregnancy outcomes. However, they may also stem from reporting discrepancies shaped by cultural norms around pregnancy recognition and stillbirth disclosure, access to clinical detection, the legal context of abortion services, and biases from interviewer effects.^25^

Heterogeneity in reported pregnancy outcomes translate into considerable differences in the magnitude of bias introduced by using a live birth denominator, and the effect of using an alternative denominator. Replacing live births with total births reduced the MMR and PRMR by up to 2.8% (Cote d'Ivoire 2021). Replacing live births with total pregnancies reduced the MMR and PRMR by up to 23% (Cambodia 2021). Given the likelihood of under-reporting of pregnancies not ending in a live birth, these figures likely underestimate the true extent of bias from using a live birth denominator.

With five years remaining in the SDG period, our findings encourage reflection on how maternal health metrics are defined and exploration of the implications of current measurement practices. While many fundamental challenges affect maternal mortality surveillance, most attention has focused on problems with the numerator, including misclassification and incompleteness of maternal deaths within CRVS systems.^26^ These issues are serious and persistent. Yet, the choice of denominator—and its effect on how maternal risk is understood—has received less scrutiny.

Any change to the denominator requires careful consideration of what is epidemiologically appropriate, the current state of data capture for pregnancy outcomes, and the implications of changes in methodology on maternal policy and programmes. Epidemiologically, a total pregnancy denominator would reflect the true population at risk. Indeed, this may constitute a long-term goal of maternal metrics. However, data limitations and reporting biases hinder its implementation in most countries in the near future.

Our findings reflect these challenges. Reported induced abortion rates in the FPH ranged from under 1% in Tanzania (2022), where abortion is highly restricted, to 14% in Cambodia (2021), where it is legal on request. These patterns align with known reporting biases in restrictive legal environments.^27^ DHS design further downward biases estimates; in ever married samples, abortions among never-married women are effectively excluded.^28^ Yet these pregnancies, often socially stigmatised, may be most likely to end in abortion. Interviewer effects further exacerbate underreporting, explaining between 0.2% and 50% of the variance in reported abortion histories.^25^

Similarly, we find reported miscarriage rates in the FPH range from 6.3% in Burkina Faso (2021) to 18% in Cambodia (2021), broadly consistent with estimates from high-income settings, where around 15% of clinically recognised pregnancies end in miscarriage.^29^ Yet, early losses, especially in the first trimester, often go undetected or unreported.^30^ These problems are likely more pronounced in low-income settings,^30^ where access to- and utilisation of-early antenatal care is substantially lower, limiting clinical detection.^31^ Adolescents and unmarried women, excluded from ever-married DHS samples, may be particularly unlikely to disclose losses, due to heightened fear of stigma from disclosure.^32^

On balance, our findings highlight the value of renewed consideration of the denominator issue and continued exploration into the feasibility of including stillbirths.^16^^,^^33^ From a methodological standpoint, a total birth denominator is more conceptually coherent, reduces incongruence with the numerator, and strengthens the validity of maternal health metrics. This introduces additional heterogeneity from both true variation in stillbirth prevalence and differences in reporting practices. However, by explicitly including stillbirths, using a total birth denominator helps reduce bias from the differential misclassification of stillbirths and early neonatal deaths in survey data, the magnitude of which varies across settings. Misclassification may occur unintentionally–especially for intrapartum-related deaths where signs of life are limited (e.g., birth asphyxia)–or intentionally, to avoid blame or clinical audit.^34^

Recent improvements in global estimation of stillbirths mean using a total birth denominator is an increasingly viable option. Although stillbirths are estimated with variable accuracy across countries, there is growing momentum to improve their measurement, particularly to track progress against the Every Newborn Action Plan target to reduce stillbirths to below 12 per 1000 total births by 2030.^13^ The WHO and United Nations currently generate stillbirth estimates from 28 weeks’ gestation,^12^ which could be used to calculate the MMR using a total birth denominator. These estimates incorporate adjustments for known biases in stillbirth data coverage, underreporting and misclassification, particularly in settings with limited or variable data quality.^12^ Given that live birth estimates in countries with poor CRVS are already primarily modelled from household survey data, this approach would be consistent with, and extend, current practices in maternal health estimation.

Including stillbirths in maternal health metrics could also help catalyse improvements in stillbirth reporting, particularly in low-income settings. Global indicators shape what is measured, tracked, and prioritised by countries and donors.^10^^,^^11^ Making stillbirths a core component of maternal metrics may increase demand for timely, accurate data and incentivise national investment in surveillance systems. This includes strengthening CRVS, where coverage remains limited and many countries lack a formal stillbirth or foetal death register.^35^ Progress will require stronger legal mandates for stillbirth review,^14^ integration into Maternal and Perinatal Death Surveillance and Response systems,^14^ and systematic facility-based registration.^35^

Crucially, improving stillbirth surveillance is not only a matter of better metrics—it is also clinically relevant. Enhanced reporting can help identify when and where stillbirths occur, which is essential for targeting improvements in the quality of late antenatal and intrapartum care.^36^

Stillbirths also represent a vitally important, yet often neglected, pregnancy outcome within the maternal health agenda. They are closely associated with obstetric complications and the quality of antepartum and intrapartum care.^12^ Grief, stigma, and social isolation associated with stillbirths can have profound consequences for women and their partners’ psychosocial wellbeing,^12^^,^^37^ as is also the case with earlier pregnancy losses.^38^ Yet stillbirths have historically received less political traction and funding than maternal deaths. More explicitly integrating stillbirths into maternal health advocacy may help elevate stillbirth prevention on the global agenda.

Despite the advantages of adopting a denominator of total births, any revision to the definition of a metric inevitably raises concerns about comparability with previous measurements. Nevertheless, methodologies should be revisited as progress is made in the availability of stillbirth data. Global estimation efforts, such as UN and WHO Joint Agency maternal mortality estimates, routinely evolve to reflect improved data and methods.^2^ As part of this progression, the upcoming 2027 and 2029 rounds of Joint Agency estimates could explore the use of a total birth denominator alongside live births, offering a valuable opportunity to assess its feasibility ahead of the post-SDG era. Any change would require careful communication among policy makers to ensure clarity in interpretation. To minimise confusion, a revised metric may also require a new label—such as MMR + or Total MMR—warranting further discussion and UN engagement.

Maternal health has historically been deprioritised on the global agenda, in part because of the small absolute numbers of deaths.^10^ As the primary indicator for SDG 3.1, a reduction in the MMR resulting from an expansion in the denominator should not be misinterpreted as justification to defund or deprioritise maternal initiatives. On the contrary, stagnating progress in improving maternal survival in recent years emphasises the urgent need to redouble our efforts to improve maternal outcomes.^2^ Therefore, any change to methodology must be made carefully, with strong country engagement, and accompanied by continued commitment to improving maternal health outcomes beyond the SDG era.

This study poses both strengths and limitations. A major strength of DHS data is their cross-national comparability, large sample sizes, and inclusion of full reproductive histories, which enable the estimation of maternal mortality and pregnancy outcomes in settings where CRVS systems are weak or incomplete, and the burden of maternal mortality is typically highest.^21^^,^^39^ This facilitated our cross-country comparable analyses of how different denominators affect maternal health metrics.

However, our reliance on DHS sibling survival data introduces well-documented limitations, including underreporting of siblings and sibling deaths, assumptions of independence in sibling mortality risk, and substantial missing data on pregnancy status, particularly for early pregnancy losses.^40^ These all may create downward bias in mortality estimates. The DHS definition of maternal mortality also departs from ICD standards by including incidental deaths, which may lead to upwardly biased estimates. Nonetheless, these limitations primarily affect the numerator and are unlikely to bias comparisons across denominators.

By contrast, underreporting and misclassification of stillbirths, miscarriages, and induced abortions directly affect the construction of denominators. These outcomes are systematically underreported, meaning that our assessment of the change in the MMR when using a total births or pregnancies denominator is likely an underestimate. Cross-country differences in underreporting introduces additional heterogeneity into alternative denominators that may bias comparisons. Further research on the extent of stillbirth underreporting by country is needed to check the validity of estimates and inform a future transition to a total birth denominator. Moreover, outdated DHS surveys and limited country coverage constrain generalisability. Disaggregated estimates of induced abortions and miscarriages were only possible in DHS-VIII surveys using the FPH module, currently available for just four countries.

As the SDG period draws to a close, the maternal health community should consider moving towards including both live births and stillbirths in the denominator of maternal health metrics in a post-SDG era. When communicated clearly, this could support and strengthen existing methods, signifying an important step towards a more inclusive and coherent understanding of maternal risk.

## Contributors

UG conceived the idea of the study, ran the database searches, developed the code, and drafted the initial manuscript.

HES built the code repository, developed the code, and drafted the initial manuscript.

HB, LL, JW, WG and VF supported the refinement of the study design, the interpretation of results, and revised the article. UG and HES accessed and verified the data. All authors had full access to all the data in the study and had final responsibility to submit for publication.

## Data sharing statement

All DHS data are available upon registration from DHS program: https://dhsprogram.com/.

Original *DHS.rates* code is open access from: https://github.com/cran/DHS.rates.

All our code used to re-calculate the sensitivity of maternal metrics is available at: https://github.com/ursulagazeley/maternal-denominators-public.git.

## Declaration of interests

We declare no competing interests.
