# Supplementary material for: Accounting for stillbirths in maternal health metrics: a cross-country analysis
Source: eClinicalMedicine. 2025 Jun 23;85:103303. doi: 10.1016/j.eclinm.2025.103303 (PMC12226388; doi:10.1016/j.eclinm.2025.103303)
Supplement: Appendix Figs. S1–S5 and Tables S1–S3 [file mmc1.docx]

**Supplementary Material**

**Accounting for stillbirths in maternal health metrics: a cross-country systematic analysis**

Ursula Gazeley^1*^, Hallie Eilerts-Spinelli^2*^, Joshua Wilde^1,3^, Hannah Blencowe^4^, Li Liu^2,5^, Wendy Graham^4^, and Veronique Filippi^4^

^1^ Leverhulme Centre for Demographic Science, Nuffield Department of Population Health, University of Oxford, Oxford, United Kingdom

^2^ Department of International Health, Johns Hopkins Bloomsberg School of Public Health, Baltimore, Maryland, United States

^3^ Portland State University, Portland, Oregon, United States

^4^ Department of Infectious Disease Epidemiology and International Health, London School of Hygiene and Tropical Medicine, London, United Kingdom

^5^ Department of Population Family and Reproductive Health, Johns Hopkins Bloomsberg School of Public Health, Baltimore, Maryland, United States

^*^ These authors contributed equally to this manuscript.

Corresponding author: [Ursula.gazeley@ndph.ox.ac.uk](mailto:Ursula.gazeley@ndph.ox.ac.uk)

# Search strategy

Embase 1974 to present

Global Health 1973 to 2025 Week 17

Medline (Ovid MEDLINE® Epub Ahead of Print, In-Process & Other Non-Indexed Citations, Ovid MEDLINE® Daily and Ovid MEDLINE®) 1946 to present

Search ran on 30.04.2025

| **No.** | **Search terms** | **Results** |
| --- | --- | --- |
| 1 | ("maternal mortality ratio" or "pregnancy-related mortality ratio" or "lifetime risk of maternal death" or "lifetime risk of pregnancy-related death" or "maternal near miss ratio" or "severe maternal outcome ratio" or "caesarean section rate" or "skilled attendance at birth rate" or "antenatal care rate" or "lifetime risk of maternal near miss" or "lifetime risk of severe maternal outcome" or "maternal metrics").ti,ab | 7855 |
| 2 | ("denominator" or "numerator" or "measurement").ti,ab. | 1560669 |
| 3 | ("live birth" or "stillbirth" or "total birth" or "pregnanc*" or "miscarriage" or "early pregnancy loss" or "spontaneous abortion" or "induced abortion" or "termination").ti,ab. | 1539114 |
| 4 | 1 and 2 and 3 | 82 |
| 5 | Remove duplicates from 4 | 43 |

# 2. Demographic and Health Survey (DHS) Sample

**Table S1 Sample of DHS data included in analyses**

| **Country** | **Year** | **DHS Phase** | **Ever-married sample** | **Repro. calendar** | **FPH** | **Calculate MMR** | **Calculate PRMR** |
| --- | --- | --- | --- | --- | --- | --- | --- |
| Afghanistan | 2015 | 7 | Yes | Yes | No | Yes | Yes |
| Angola | 2015 | 7 | No | Yes | No | Yes | Yes |
| Benin | 2017 | 7 | No | Yes | No | Yes | Yes |
| Bolivia | 2008 | 5 | No | Yes | No | No | Yes |
| Brazil | 1996 | 3 | No | Yes | No | No | Yes |
| Burkina Faso | 2021 | 8 | No | Yes | Yes | Yes | Yes |
| Burundi | 2016 | 7 | No | Yes | No | Yes | Yes |
| Cambodia | 2021 | 8 | No | Yes | Yes | Yes | Yes |
| Colombia | 2015 | 7 | No | Yes | No | Yes | Yes |
| Comoros | 2012 | 6 | No | Yes | No | No | Yes |
| Côte d’Ivoire | 2021 | 8 | No | Yes | Yes | Yes | Yes |
| Dominican Republic | 2002 | 4 | No | Yes | No | No | Yes |
| Eswatini | 2006 | 5 | No | Yes | No | No | Yes |
| Ethiopia | 2016 | 7 | No | Yes | No | No | Yes |
| Gabon | 2019 | 7 | No | Yes | No | Yes | Yes |
| Gambia | 2019 | 7 | No | Yes | No | Yes | Yes |
| Guatemala | 2015 | 6 | No | Yes | No | No | Yes |
| Guinea | 2005 | 4 | No | Yes | No | No | Yes |
| Indonesia | 2012 | 6 | No | Yes | No | No | Yes |
| Jordan | 1997 | 3 | Yes | Yes | No | No | Yes |
| Kenya | 2014 | 6 | No | Yes | No | No | Yes |
| Lesotho | 2014 | 6 | No | Yes | No | No | Yes |
| Liberia | 2019 | 7 | No | Yes | No | Yes | Yes |
| Madagascar | 2008 | 5 | No | Yes | No | No | Yes |
| Malawi | 2015 | 7 | No | Yes | No | Yes | Yes |
| Mali | 2018 | 7 | No | Yes | No | Yes | Yes |
| Mauritania | 2020 | 7 | No | Yes | No | Yes | Yes |
| Morocco | 2003 | 4 | No | Yes | No | No | Yes |
| Mozambique | 2022 | 8 | No | Yes | No | Yes | Yes |
| Myanmar (Burma) | 2016 | 7 | No | Yes | No | No | Yes |
| Namibia | 2013 | 6 | No | Yes | No | No | Yes |
| Nepal | 2016 | 7 | No | Yes | No | Yes | Yes |
| Niger | 2012 | 6 | No | Yes | No | No | Yes |
| Nigeria | 2018 | 7 | No | Yes | No | Yes | Yes |
| Papua New Guinea | 2017 | 7 | No | Yes | No | Yes | Yes |
| Peru | 2011 | 6 | No | Yes | No | No | Yes |
| Philippines | 1998 | 3 | No | Yes | No | No | Yes |
| Rwanda | 2019 | 7 | No | Yes | No | Yes | Yes |
| Senegal | 2023 | 8 | No | Yes | No | Yes | Yes |
| Sierra Leone | 2019 | 7 | No | Yes | No | Yes | Yes |
| South Africa | 2016 | 7 | No | Yes | No | Yes | Yes |
| Tanzania | 2022 | 8 | No | Yes | Yes | Yes | Yes |
| Timor-Leste | 2016 | 7 | No | Yes | No | Yes | Yes |
| Uganda | 2016 | 7 | No | Yes | No | Yes | Yes |
| Zambia | 2018 | 7 | No | Yes | No | Yes | Yes |
| Zimbabwe | 2015 | 7 | No | Yes | No | No | Yes |

# 3. Reported pregnancy outcomes for Full Pregnancy History

**Figure S1 Reported pregnancy outcomes for surveys using Full Pregnancy History**

**
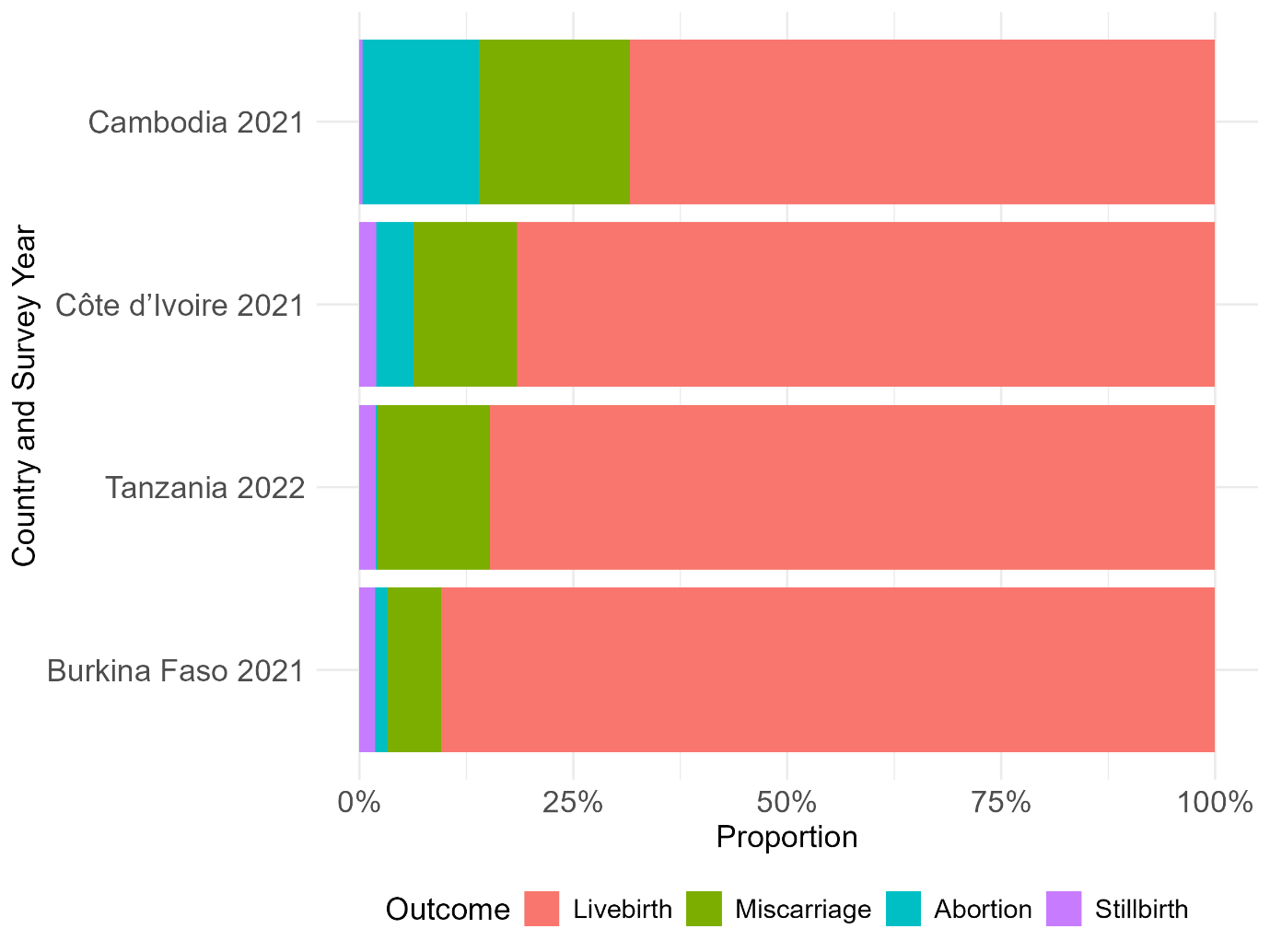
**

# 4. Maternal Mortality Ratio with total pregnancy denominator


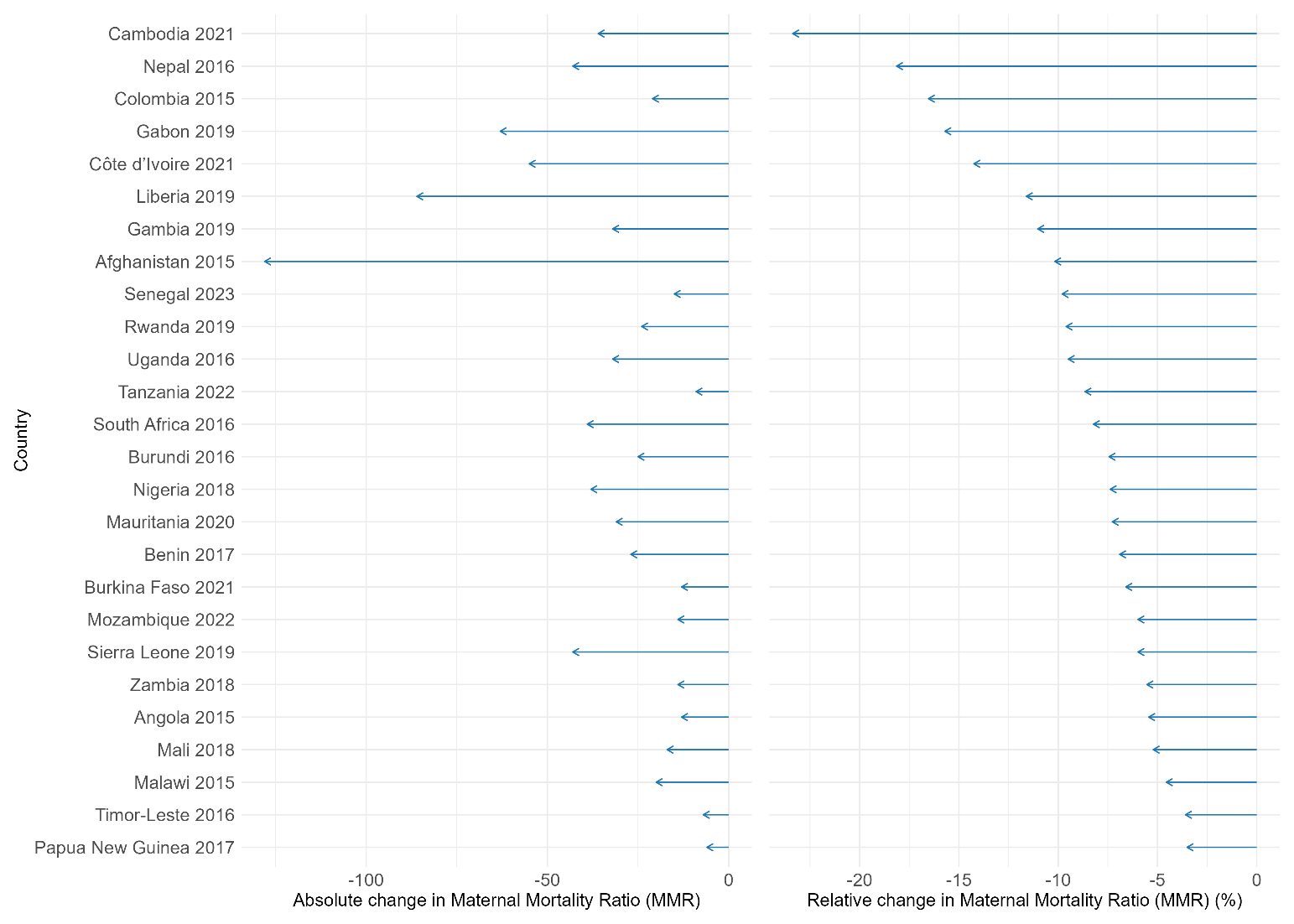
**Figure S2 Absolute and relative change in Maternal Mortality Ratio (MMR) using a total pregnancy denominator instead of live births**

# 5. Pregnancy-Related Mortality Ratio with total pregnancy denominator

**
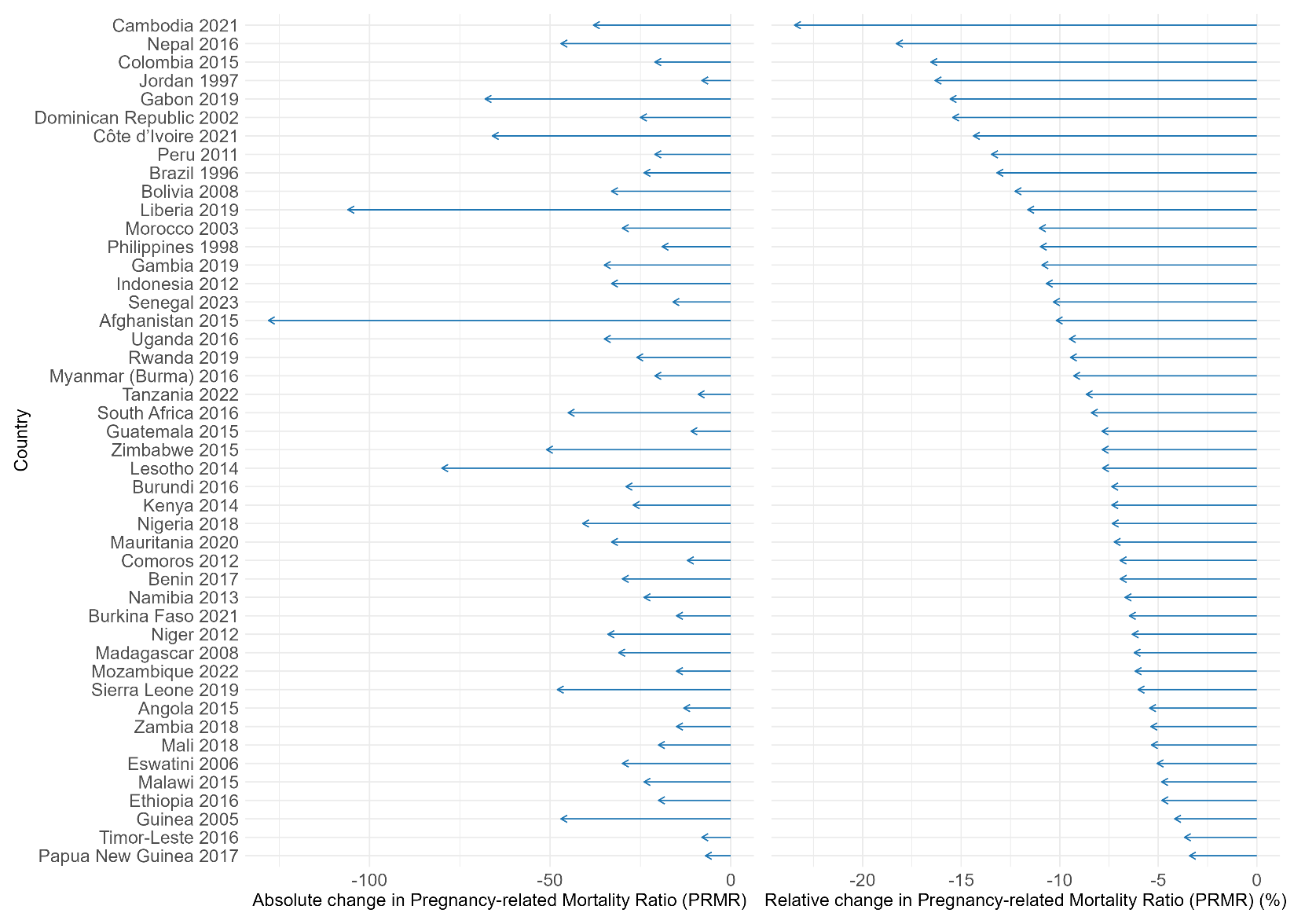
Figure S3 Absolute and relative change in Pregnancy-related Mortality Ratio (PRMR) using a total pregnancy denominator instead of live births**

# 6. Full results

**Table S2 MMR using total birth or total pregnancy denominators**

|  | | | | | | | |
| --- | --- | --- | --- | --- | --- | --- | --- |
|  |  | **Total birth denominator** | | | **Total pregnancy denominator** | | |
| Country year | MMR (live births) | MMR re-calculated | Absolute change in MMR | Relative change in MMR (%) | MMR recalculated | Absolute change in MMR | Relative change in MMR (%) |
| Afghanistan 2015 | 1258 | 1233 | -25 | -2.0 | 1130 | -128 | -10.2 |
| Angola 2015 | 239 | 238 | -1 | -0.4 | 226 | -13 | -5.4 |
| Benin 2017 | 391 | 387 | -4 | -1.0 | 364 | -27 | -6.9 |
| Burkina Faso 2021 | 197 | 195 | -2 | -1.0 | 184 | -13 | -6.6 |
| Burundi 2016 | 336 | 329 | -7 | -2.1 | 311 | -25 | -7.4 |
| Cambodia 2021 | 154 | 152 | -2 | -1.3 | 118 | -36 | -23.4 |
| Colombia 2015 | 127 | 125 | -2 | -1.6 | 106 | -21 | -16.5 |
| Côte d’Ivoire 2021 | 386 | 375 | -11 | -2.8 | 331 | -55 | -14.2 |
| Gabon 2019 | 401 | 394 | -7 | -1.7 | 338 | -63 | -15.7 |
| Gambia 2019 | 290 | 284 | -6 | -2.1 | 258 | -32 | -11.0 |
| Liberia 2019 | 741 | 731 | -10 | -1.3 | 655 | -86 | -11.6 |
| Malawi 2015 | 439 | 434 | -5 | -1.1 | 419 | -20 | -4.6 |
| Mali 2018 | 326 | 322 | -4 | -1.2 | 309 | -17 | -5.2 |
| Mauritania 2020 | 426 | 421 | -5 | -1.2 | 395 | -31 | -7.3 |
| Mozambique 2022 | 234 | 231 | -3 | -1.3 | 220 | -14 | -6.0 |
| Nepal 2016 | 237 | 235 | -2 | -0.8 | 194 | -43 | -18.1 |
| Nigeria 2018 | 515 | 503 | -12 | -2.3 | 477 | -38 | -7.4 |
| Papua New Guinea 2017 | 171 | 170 | -1 | -0.6 | 165 | -6 | -3.5 |
| Rwanda 2019 | 250 | 246 | -4 | -1.6 | 226 | -24 | -9.6 |
| Senegal 2023 | 153 | 151 | -2 | -1.3 | 138 | -15 | -9.8 |
| Sierra Leone 2019 | 719 | 709 | -10 | -1.4 | 676 | -43 | -6.0 |
| South Africa 2016 | 474 | 469 | -5 | -1.1 | 435 | -39 | -8.2 |
| Tanzania 2022 | 104 | 102 | -2 | -1.9 | 95 | -9 | -8.7 |
| Timor-Leste 2016 | 195 | 195 | 0 | 0.0 | 188 | -7 | -3.6 |
| Uganda 2016 | 337 | 332 | -5 | -1.5 | 305 | -32 | -9.5 |
| Zambia 2018 | 253 | 250 | -3 | -1.2 | 239 | -14 | -5.5 |

**Table S3 PRMR using total birth or total pregnancy denominators**

|  |  | **Total birth denominator** | | | **Total pregnancy denominator** | | |
| --- | --- | --- | --- | --- | --- | --- | --- |
| Country year | PRMR (live births) | PRMR re-calculated | Absolute change in PRMR | Relative change in PRMR (%) | PRMR re-calculated | Absolute change in PRMR | Relative change in PRMR (%) |
| Afghanistan 2015 | 1258 | 1233 | -25 | -2.0 | 1130 | -128 | -10.2 |
| Angola 2015 | 239 | 238 | -1 | -0.4 | 226 | -13 | -5.4 |
| Benin 2017 | 433 | 429 | -4 | -0.9 | 403 | -30 | -6.9 |
| Bolivia 2008 | 269 | 266 | -3 | -1.1 | 236 | -33 | -12.3 |
| Brazil 1996 | 182 | 180 | -2 | -1.1 | 158 | -24 | -13.2 |
| Burkina Faso 2021 | 232 | 229 | -3 | -1.3 | 217 | -15 | -6.5 |
| Burundi 2016 | 394 | 386 | -8 | -2.0 | 365 | -29 | -7.4 |
| Cambodia 2021 | 162 | 160 | -2 | -1.2 | 124 | -38 | -23.5 |
| Colombia 2015 | 127 | 125 | -2 | -1.6 | 106 | -21 | -16.5 |
| Comoros 2012 | 173 | 172 | -1 | -0.6 | 161 | -12 | -6.9 |
| Côte d’Ivoire 2021 | 459 | 446 | -13 | -2.8 | 393 | -66 | -14.4 |
| Dominican Republic 2002 | 162 | 160 | -2 | -1.2 | 137 | -25 | -15.4 |
| Eswatini 2006 | 593 | 584 | -9 | -1.5 | 563 | -30 | -5.1 |
| Ethiopia 2016 | 415 | 410 | -5 | -1.2 | 395 | -20 | -4.8 |
| Gabon 2019 | 437 | 429 | -8 | -1.8 | 369 | -68 | -15.6 |
| Gambia 2019 | 321 | 314 | -7 | -2.2 | 286 | -35 | -10.9 |
| Guatemala 2015 | 140 | 139 | -1 | -0.7 | 129 | -11 | -7.9 |
| Guinea 2005 | 1127 | 1103 | -24 | -2.1 | 1080 | -47 | -4.2 |
| Indonesia 2012 | 309 | 305 | -4 | -1.3 | 276 | -33 | -10.7 |
| Jordan 1997 | 49 | 49 | 0 | 0.0 | 41 | -8 | -16.3 |
| Kenya 2014 | 367 | 362 | -5 | -1.4 | 340 | -27 | -7.4 |
| Lesotho 2014 | 1022 | 1003 | -19 | -1.9 | 942 | -80 | -7.8 |
| Liberia 2019 | 912 | 900 | -12 | -1.3 | 806 | -106 | -11.6 |
| Madagascar 2008 | 498 | 492 | -6 | -1.2 | 467 | -31 | -6.2 |
| Malawi 2015 | 497 | 491 | -6 | -1.2 | 473 | -24 | -4.8 |
| Mali 2018 | 374 | 369 | -5 | -1.3 | 354 | -20 | -5.3 |
| Mauritania 2020 | 456 | 451 | -5 | -1.1 | 423 | -33 | -7.2 |
| Morocco 2003 | 272 | 269 | -3 | -1.1 | 242 | -30 | -11.0 |
| Mozambique 2022 | 243 | 240 | -3 | -1.2 | 228 | -15 | -6.2 |
| Myanmar (Burma) 2016 | 226 | 223 | -3 | -1.3 | 205 | -21 | -9.3 |
| Namibia 2013 | 359 | 356 | -3 | -0.8 | 335 | -24 | -6.7 |
| Nepal 2016 | 257 | 254 | -3 | -1.2 | 210 | -47 | -18.3 |
| Niger 2012 | 538 | 530 | -8 | -1.5 | 504 | -34 | -6.3 |
| Nigeria 2018 | 559 | 547 | -12 | -2.1 | 518 | -41 | -7.3 |
| Papua New Guinea 2017 | 205 | 204 | -1 | -0.5 | 198 | -7 | -3.4 |
| Peru 2011 | 156 | 156 | 0 | 0.0 | 135 | -21 | -13.5 |
| Philippines 1998 | 173 | 170 | -3 | -1.7 | 154 | -19 | -11.0 |
| Rwanda 2019 | 275 | 271 | -4 | -1.5 | 249 | -26 | -9.5 |
| Senegal 2023 | 155 | 153 | -2 | -1.3 | 139 | -16 | -10.3 |
| Sierra Leone 2019 | 797 | 786 | -11 | -1.4 | 749 | -48 | -6.0 |
| South Africa 2016 | 536 | 530 | -6 | -1.1 | 491 | -45 | -8.4 |
| Tanzania 2022 | 104 | 102 | -2 | -1.9 | 95 | -9 | -8.7 |
| Timor-Leste 2016 | 218 | 218 | 0 | 0.0 | 210 | -8 | -3.7 |
| Uganda 2016 | 368 | 362 | -6 | -1.6 | 333 | -35 | -9.5 |
| Zambia 2018 | 279 | 276 | -3 | -1.1 | 264 | -15 | -5.4 |
| Zimbabwe 2015 | 650 | 641 | -9 | -1.4 | 599 | -51 | -7.8 |

# 7. Uncertainty analysis


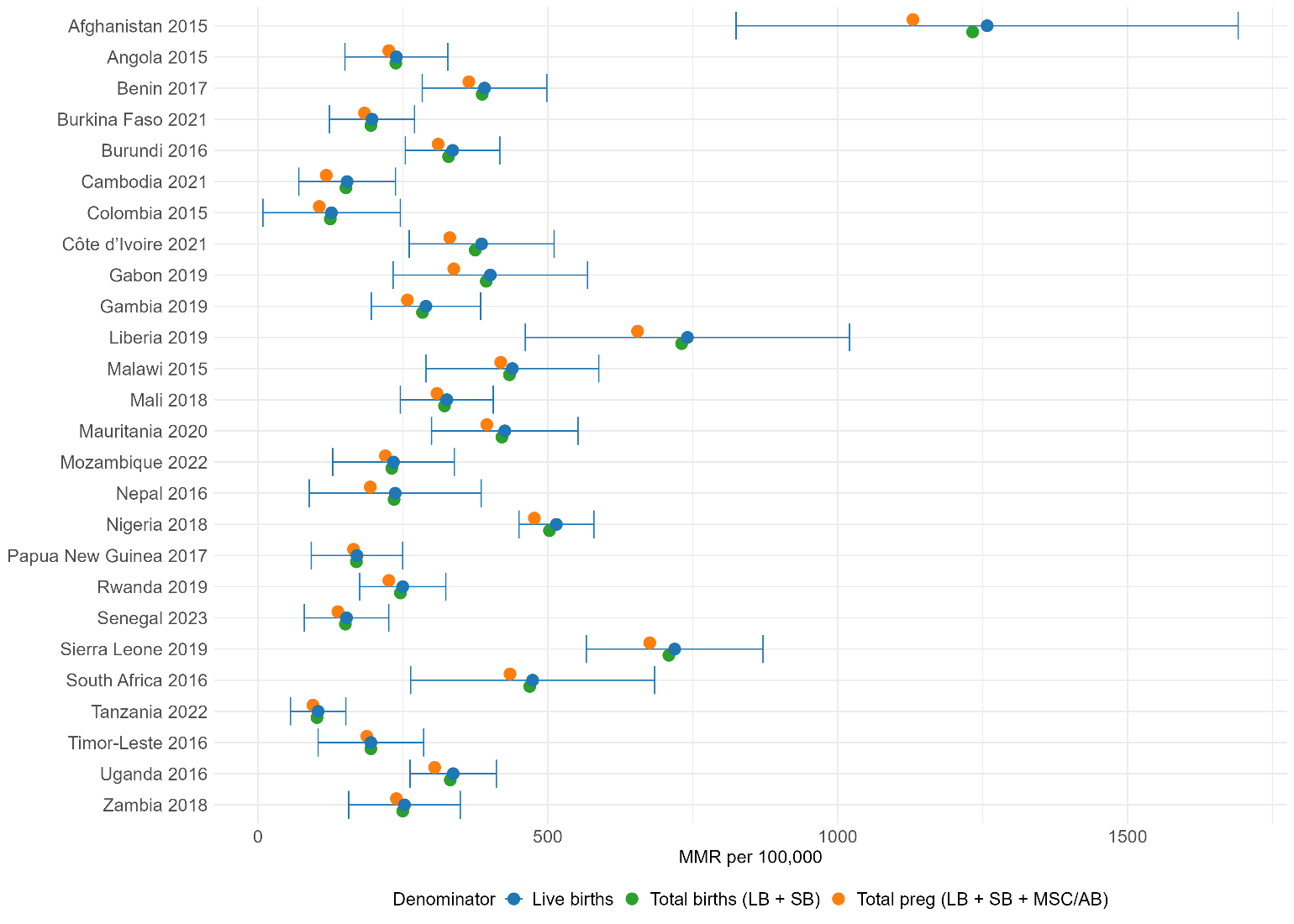
**Figure S4 Estimates and 95% confidence intervals for unadjusted Maternal Mortality Ratio (MMR) alongside estimates that were re-calculated using a total birth or total pregnancy denominator instead of live births**

Note: 95% confidence intervals calculated for unadjusted estimates (live births denominator) only.


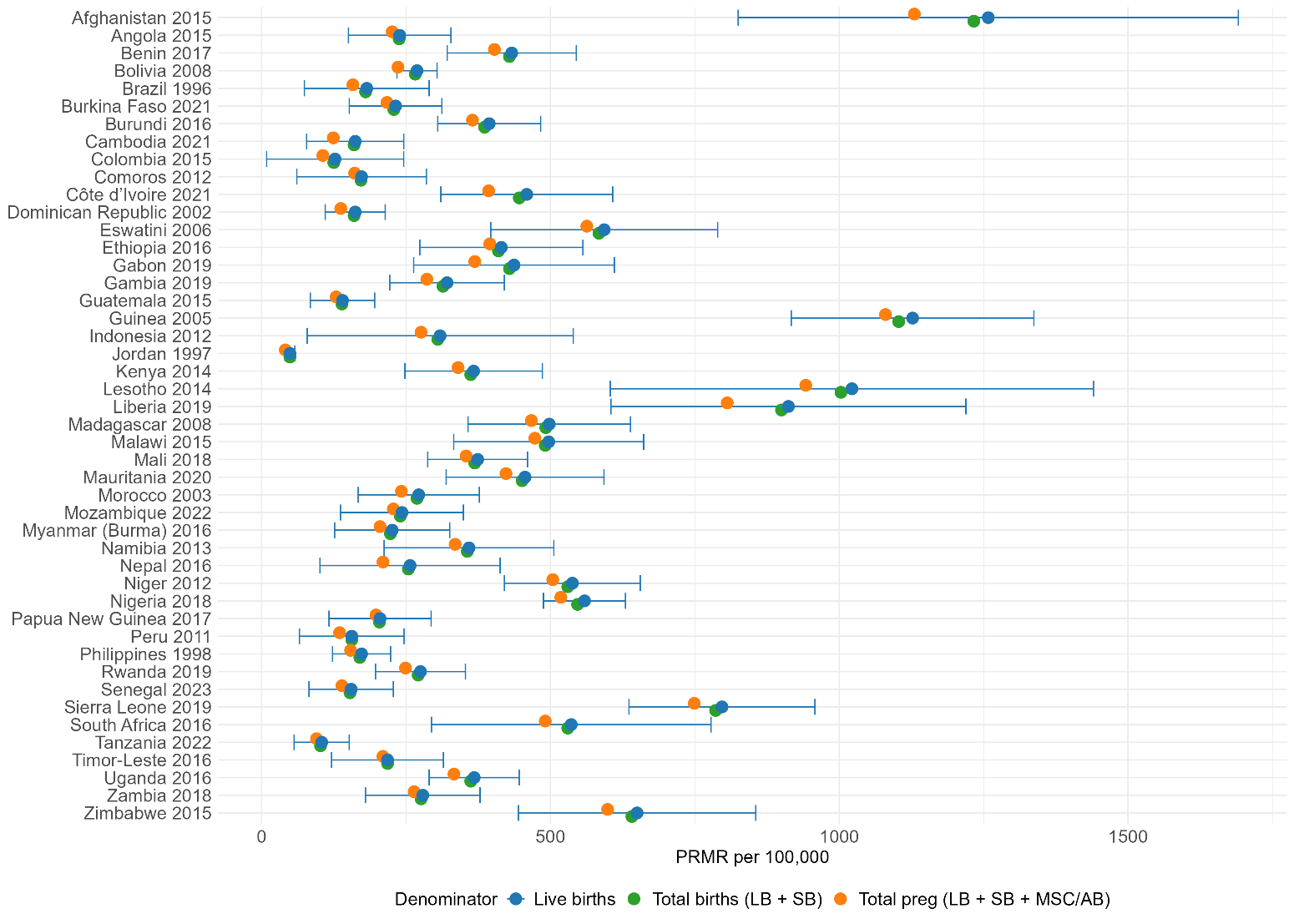
**Figure S5 Estimates and 95% confidence intervals for unadjusted Pregnancy-related Mortality Ratio (PRMR) alongside estimates that were re-calculated using a total birth or total pregnancy denominator instead of live births**

Note: 95% confidence intervals calculated for unadjusted estimates (live births denominator) only.
